# In this issue of *Current Oncology*

**Published:** 2008-06

**Authors:** P. Gold

This issue of *Current Oncology* cuts a broad swath through highly topical subjects in cancer biology and cancer care. An article by Chasen and Dippenaar addresses the ever-increasing emphasis on dealing with cancer as a systemic disease, showing how to assure optimal care, the patient’s condition must be addressed in a multidisciplinary fashion, including dealing with nutritional concerns, rehabilitation, and psychic distress.

Batist and Shinder give a fascinating description of the growth—and changes—in the McGill University Department of Oncology since the start of the 1990s. They note how the functional alterations during the time since have largely been driven by the evolution of knowledge in the field of oncology.

Two papers address aspects of gynecologic cancer. Nayot *et al.* describe a pilot study indicating that preoperative positron-emission tomography with computed tomography does not necessarily predict lymph node status in endometrial cancer. Then Gien and associates present evidence that hormonal therapy may be of no value as adjuvant treatment for patients with stage I endometrial cancer.

Two articles consider differing aspects of targeted biopharmaceutical—or perhaps “bioceutical”—therapy. Examining the cost burden of trastuzumab and bevacizumab (two monoclonal antibodies) as therapy for solid tumours in Canada, Drucker *et al.* effectively raise the question, “When we finally have a variety of cures for various cancers requiring this form of targeted therapy, will Canada’s publicly funded health care system be able to afford them?” In the same domain, but with a different spin, Trinkaus and colleagues report on a patient with drug-induced immune thrombocytopenia secondary to sunitinib, a specific inhibitor of a tyrosine kinase. Theirs is the first such report in the literature.

In an international submission, Amouzegar–Hashemi *et al.* show that the outcomes for single as compared with multiple fractions of palliative radio-therapy for bone metastases in Iranian patients are similar to those reported elsewhere in the literature. And finally, *Current Oncology* is pleased to present a series of abstracts from the Ontario Thoracic Cancer Conference that provide significant additions to knowledge in this area of activity.

**Announcing new additions to the Current Oncology Editorial Board**

Current Oncology is pleased to welcome the following esteemed collegues to the editorial board:

R. Daniel Bonfil PhD

Wen G. Jiang MD PhD

Gurmit Singh PhD

Shigetoyo Saji MD PhD

Rolf Lamerz MD, Professor Emeritus

Francesco M. Marincola MD PhD

David Goldenberg ScD MD

Susan Slovin PhD MD

